# STPP-UP: An alternative method for drug target identification using protein thermal stability

**DOI:** 10.1016/j.jbc.2023.105279

**Published:** 2023-09-22

**Authors:** Dick W. Zijlmans, Miguel Hernández-Quiles, Pascal W.T.C. Jansen, Isabelle Becher, Frank Stein, Mikhail M. Savitski, Michiel Vermeulen

**Affiliations:** 1 Department of Molecular Biology, Faculty of Science, Radboud Institute for Molecular Life Sciences, Oncode Institute, Radboud University Nijmegen, Nijmegen, The Netherlands; 2 Division of Molecular Genetics, The Netherlands Cancer Institute, Amsterdam, The Netherlands; 3 European Molecular Biology Laboratory, Genome Biology Unit, Heidelberg, Germany; 4 Proteomics Core Facility, European Molecular Biology Laboratory, Heidelberg, Germany

**Keywords:** thermal proteomics, STPP-UP, drug discovery, protein stability, TPP

## Abstract

Thermal proteome profiling (TPP) has significantly advanced the field of drug discovery by facilitating proteome-wide identification of drug targets and off-targets. However, TPP has not been widely applied for high-throughput drug screenings, since the method is labor intensive and requires a lot of measurement time on a mass spectrometer. Here, we present Single-tube TPP with Uniform Progression (STPP-UP), which significantly reduces both the amount of required input material and measurement time, while retaining the ability to identify drug targets for compounds of interest. By using incremental heating of a single sample, changes in protein thermal stability across a range of temperatures can be assessed, while alleviating the need to measure multiple samples heated to different temperatures. We demonstrate that STPP-UP is able to identify the direct interactors for anticancer drugs in both human and mice cells. In summary, the STPP-UP methodology represents a useful tool to advance drug discovery and drug repurposing efforts.

Advances in the field of drug discovery have provided a wide range of therapies for a multitude of diseases. However, a major issue of concern is the fact that many drugs show significant off-target effects in patients, resulting in unfavorable prognosis and unwanted side-effects. Thermal proteome profiling (TPP) was originally developed as a method to identify the direct and indirect targets of newly developed drugs (1, 2), thereby potentially reducing the failure rate of compounds during different stages of drug discovery. TPP measures proteome-wide changes in thermal stability between any two conditions and uses that to infer a gain or loss of interactions, that is proteins that interact with a particular compound will show an increased stability and will be more resistant to thermal denaturation (Fig. S1*A*). Alternatively, compounds that inhibit a certain protein can induce loss of another protein’s normal targets or substrates, resulting in decreased thermal stability (3).

In a conventional TPP workflow, cells are treated with a compound of interest for a certain amount of time (Fig. 1*A*). Cells are then harvested, divided into multiple samples, and exposed to a distinct temperature for a fixed amount of time. Cells are then lysed, after which the lysate is centrifuged and filtered to remove denatured proteins. Equal amounts of filtered lysates for each temperature are then digested, labeled with tandem mass tag, pooled by either temperature (1D-TPP) or condition (2D-TPP (4)), and measured by mass spectrometry (MS) to generate denaturation curves for each detected protein. Shifts in denaturation curves between conditions reveal which proteins are altered in their thermal stability and are thus potentially affected by the compound of interest.

**Figure 1:**
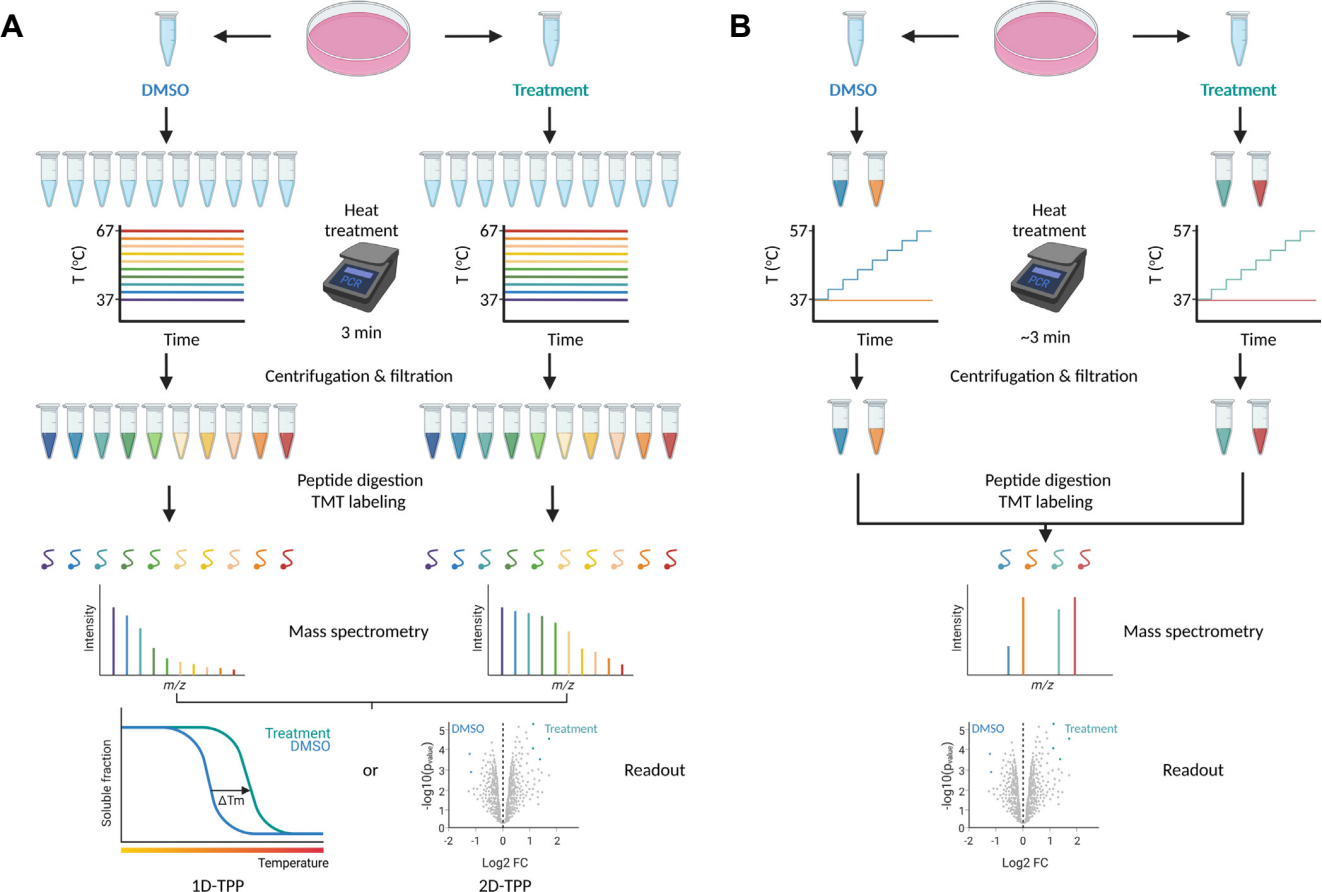
**Comparing workflows of TPP and STPP-UP.***A*, example of a TPP workflow. Cells of two different conditions are harvested and distributed over multiple tubes. Each tube is exposed to a distinct set temperature for 3 min, followed by 3 min of incubation at room temperature (RT) and lysis. Lysates are then centrifuged and filtered to remove precipitated proteins and equal volumes of filtered lysates are digested and labeled with TMT. TMT labeling can be done per temperature (1D-TPP) or replicate-condition (2D-TPP). Using 1D-TPP allows for more accurate determination of melting curves, whereas 2D-TPP allows for more accurate comparison between replicates/conditions for each temperature. After labeling, samples are pooled and analyzed by mass spectrometry. As readout, 1D-TPP uses shifts in T_m_ obtained from melting curves to assess changes in stability, whereas 2D-TPP uses stability scores to determine changes in stability. Stability scores are calculated as the sum of the log-transformed fold changes between conditions at each temperature adjusted to the relative fold changes measured at the first two temperatures. Figure was made with BioRender. *B*, example of a STPP-UP workflow. Cells of two different conditions are harvested and added to two tubes. One tube (“test”) is then exposed to an incrementally increasing temperature until a T_max_ is reached, while the other (“control”) is either exposed to 37 °C for 3 min or kept at room temperature (RT). After heating, samples are incubated at RT for 3 min and subsequently lysed. Lysates are then centrifuged and filtered to remove precipitated proteins and equal volumes of filtered lysates are digested and labeled with TMT. In STPP-UP, all replicates for both test and control samples for both conditions can be labeled together and combined into a single sample using TMTpro 16-plex. Samples are then subjected to mass spectrometry. As a readout, STPP-UP uses changes in abundance to determine changes in stability. Control samples are used to adjust the test samples for base differences in protein abundance between conditions. Figure was made with BioRender. STPP-UP, Single-tube TPP with Uniform Progression; TMT, tandem mass tag; TTP, thermal proteome profiling.

The TPP and in particular the 2D-TPP layout enable highly sensitive detection of protein–drug interactions, but these methods require a substantial amount of MS measurement time and input material (Fig. 1*A*). It is thus desirable to develop experimental formats that, while less sensitive, require less measurement time and input material, thus enabling large-scale screenings. Recently, the Proteome Integral Solubility Alteration method was developed, which reduces measurement time by pooling samples from different heat treatments into a single sample (5). PISA significantly increases sample throughput at the cost of lower sensitivity, but still requires time-consuming sample preparation (6).

## Results/discussion

We reasoned that by altering the heat treatment program such that temperature exposure, and accordingly protein denaturation, is incremental as opposed to constant, it should be possible to assess changes in protein stability between conditions in a single sample (Fig. 1*B*). This methodology, which we have termed Single-tube TPP with Uniform Progression (STPP-UP) reduces the required measurement time by a factor of 10 and the required amount of input material by at least a factor of 5, compared to conventional TPP. In STPP-UP, a single sample of cells is exposed to an incrementally increasing temperature until a T_max_ is reached, in contrast to using multiple samples, each exposed to a distinct temperature. In this manner, if the denaturation rate for a particular protein is different at any temperature between the two conditions, this will result in changes in protein abundance that will persist and potentially accumulate until T_max_ is reached (Fig. S1*B*). Optionally, a 37 °C or nonheated control sample can be included to adjust for base differences in protein abundance between conditions.

In STPP-UP, choosing the right heating conditions is essential. Protein denaturation should be such that differences between conditions can be observed, being neither too high nor too low as differences in protein abundances between the two conditions may be lost or may not yet have materialized. At higher temperatures, even a relatively short exposure time will result in substantial denaturation for the majority of the proteins (Fig. S2*A*). To determine the optimal combination of T_max_ and ramp rate for STPP-UP experiments, we analyzed public TPP datasets (7, 8, 9, 10) to determine the average T_m-50_, which is the melting temperature at which 50% of the protein is denatured relative to the lowest temperature. The average T_m-50_ is around 51 °C for mammals and is stable between species (Fig. S2*B*). Next, we measured how global protein abundances are affected in E14 mouse embryonic stem cells (mESCs) and LS 174T cells after exposing them to the TPP and STPP-UP heat gradients. We used several T_max_ and ramp rates to determine which heating conditions would be similar to the TPP T_m-50_ in terms of protein abundance (Fig. S2*C*). We determined that for both cell types, a STPP-UP T_max_ of 57 °C with a ramp rate of 0.2 °C/s is equivalent to a respective TPP T_m-50_ of ∼52 °C.

**Figure 2:**
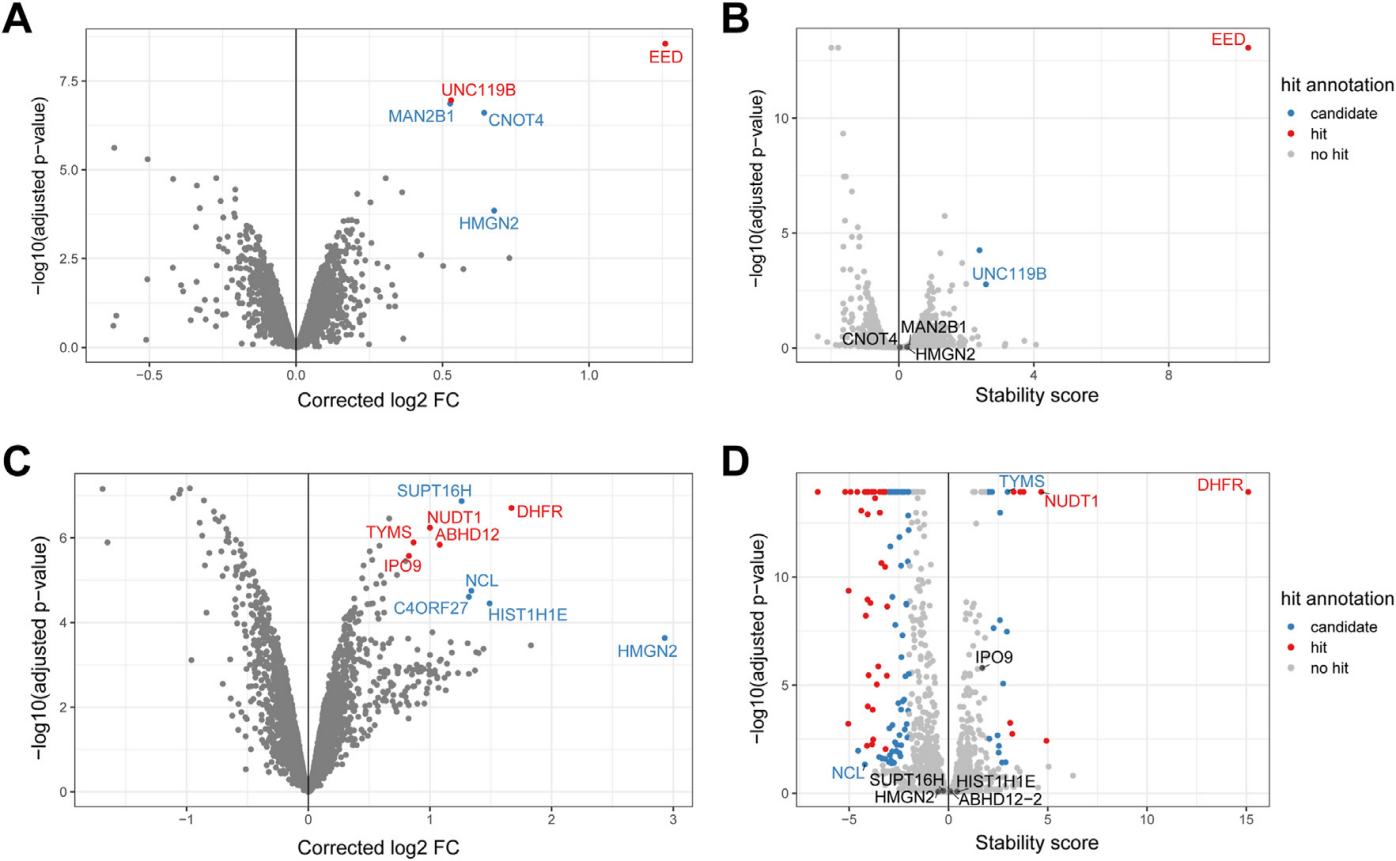
**STPP-UP identifies direct drug targets similar to TPP.***A*, *volcano plot* of STPP-UP showing changes in protein abundance between EED226- and DMSO-treated E14 mESCs after correction. EED226 treatment was done at 10 μM for 1 h. T_max_ was set at 57 °C and ramp rate at 0.2 °C/s. Three biological replicates were used per condition. Significantly enriched proteins (log2 FC > 0.5, corrected *p*-value < 0.001, students *t* test) are highlighted. Proteins showing mostly enrichment in control samples in Fig. S3*B* are highlighted in *blue*, others are highlighted in *red*. *B*, *volcano plot* of stability scores calculated from the TPP experiment in E14 mESCs. Cells were treated with 10 μM EED226 or DMSO for 1 h. Two biological replicates were used per condition. Proteins with a stability score >3 and an FDR <0.01 were designated as “hit” and are highlighted in *red*. Proteins with a stability score >2 and an FDR <0.05 were designated as “candidate” and are highlighted in *blue*. *C*, *volcano plot* of STPP-UP showing changes in protein abundance between C1- and DMSO-treated LS 174T cells after correction. C1 treatment was done at 10 μM for 1 h. T_max_ was set at 57 °C and ramp rate at 0.2 °C/s. Three biological replicates were used per condition. Several significantly enriched proteins (log2 FC > 0.5, *p*-value < 0.001, students *t* test) are highlighted. Proteins showing mostly enrichment in control samples in Fig. S5*B* are highlighted in *blue*, others are highlighted in *red*. *D*, *volcano plot* of stability scores calculated from the TPP experiment in LS 174T cells. Cells were treated with 10 μM C1 or DMSO for 1 h. Three biological replicates were used per condition. Proteins with a stability score >3 and an FDR <0.01 were designated as “hit” and are highlighted in *red*. Proteins with a stability score >2 and an FDR <0.05 were designated as “candidate” and are highlighted in *blue*. DMSO, dimethyl sulfoxide; FDR, false discovery rate; mESC, mouse embryonic stem cell; STPP-UP, Single-tube TPP with Uniform Progression; TTP, thermal proteome profiling.

To benchmark this new method, we performed STPP-UP on E14 mESC treated with the PRC2 inhibitor EED226 (11). PRC2 is a histone methyltransferase that catalyzes the methylation of H3K27me3, an important epigenetic mark that is associated with gene silencing. PRC2 consists of several core proteins, including EED, which can be specifically inhibited using EED226. We used a T_max_ set to 57 °C and a ramp rate of 0.2 °C/s and compared the results to a conventional TPP experiment (Figs. 2, *A* and *B* and S3*A*). Samples in the TPP experiment were multiplexed by condition and combined by temperature (2D-TPP (4)) to allow for the most accurate comparison between conditions at each temperature point. Stability scores were calculated as the sum of the log-transformed fold changes measured at each temperature point adjusted to the relative fold changes measured at the first two temperatures, which represent differences in base protein abundance level. We observed reduced denaturation of the EED protein at higher temperatures in the EED226-treated cells (Fig. S3*A*) and an increased stabilization of the protein in both the TPP and STPP-UP experiments (Fig. 2, *A* and *B*), confirming the direct binding of EED226 to the EED protein. We also observe slight stabilization of UNC119B in both STPP-UP and TPP experiments, which is likely an off-target effect as UNC119B has no known Polycomb-related functions. We identified several additional proteins that show stabilization in the STPP-UP experiment, which did not show altered stability in the TPP experiment. However, it should be noted that for most of these proteins, the stabilizing effect they exhibit is caused by correcting for base differences in protein abundance (Fig. S3*B*). We validated our findings in another cell line by performing STPP-UP using EED226 in R1 mESCs using different T_max_ and a ramp rate of 0.2 °C/s (Fig. S4). We observed enrichment of EED in EED226-treated cells compared to dimethyl sulfoxide (DMSO) only at a T_max_ of 57 °C, in line with our reasoning that choosing lower or higher T_max_ would result in too little or too much protein denaturation, respectively, compromising the ability to measure any differences between the treatment and control conditions.

We further validated STPP-UP in human cells, using the anticancer drug C1 (3) applied to the LS 174T colorectal cancer cell line (Figs. 2, *C* and *D* and S5*A*). In line with previously published TPP data (3), we successfully identify dihydrofolate reductase (DHFR) as the primary target for C1 using STPP-UP. While the target-to-background enrichment is not as strong as in the mouse STPP-UP experiment, and there appear to be more off-targets, these effects can mostly be attributed to correcting for abundance differences (Fig. S5*B*), as enrichment of DHFR becomes much more pronounced upon removal of these correction effects. To confirm that any combination of T_max_ and ramp rate that results in ∼50% denaturation (Fig. S2*C*) can be used for STPP-UP, we repeated the experiment using different heating conditions, with T_max_ set at 55 °C and a ramp rate of 0.1 °C/s. This again resulted in the identification of DHFR as the most stabilized protein under these conditions (Fig. S5*C*), together with the secondary (off-)target TYMS, which stabilizes due to downstream effects of DHFR inhibition (3).

Given the recent development of an isothermal shift assay (12), we next assess how incremental heating to T_max_ compared to constant incubation at T_max_. To this end, we used the TPP-validated pan-kinase inhibitor staurosporine (2) in K562 cells and combined incremental heating and constant incubation in the same tandem mass tag experiment to allow for an accurate comparison. We find that both methods identify significant thermal shifts for many proteins known to be (de)stabilized using TPP as well as proteins that are part of the global kinome (Fig. S6, *A* and *B*), with each method also identifying unique thermal shifts missed by the other. However, using incremental heating allows for the evaluation of differences in thermal stability across multiple temperature points, whereas constant incubation at a given temperature only provides a snapshot into a proteins’ melting profile, which may result in the identification of false positives (Fig. S7, *A* and *B*).

To summarize, this work reports a straightforward one-pot method to detect proteome-wide changes in thermal stability upon exposure to drugs, while requiring significantly less input material and instrument measurement time compared to conventional TPP. We applied the STPP-UP method to accurately determine the direct targets of drug compounds in both mouse and human cells. While TPP has a higher resolution, accuracy, and sensitivity compared to STPP-UP (STPP-UP will not detect stability changes for proteins with a high T_m-50_, depending on the T_max_), STPP-UP potentially allows for a vastly higher throughput. In the future, STPP-UP can therefore be used during the early phases of drug discovery to screen relatively large amounts of lead compounds. After screening with STPP-UP, the most promising hits can then be followed up on using TPP. We envision broad application for STPP-UP in the fields of drug development and cancer research.

## Experimental procedures

### Cell culture

E14 and R1 mESCs were cultured on 0.15% (w/v) gelatin-coated dishes in Dulbecco’s Modified Eagle Medium (Gibco) supplemented with 2 mM GlutaMAX (Gibco), 1 mM sodium pyruvate (Gibco), 1× nonessential amino acids (Gibco), 50 U/ml penicillin-streptomycin (Gibco), 15% fetal bovine serum (HyClone), leukemia inhibitory factor (produced in-house), and 100 μM β-mercaptoethanol (Sigma-Aldrich). LS 174T colorectal cancer cells were cultured in Roswell Park Memorial Institute 1640 medium (Gibco) supplemented with 50 U/ml penicillin-streptomycin and 10% fetal bovine serum.

### Thermal proteome profiling

TPP was done as previously described (4). In brief, cells were harvested after 1 h of treatment with the compound of interest or DMSO, washed with PBS, and counted. Ten aliquots, each containing 10^6^ cells in 100 μl PBS, were distributed in a row of a 96-well PCR plate. After centrifugation (300*g* for 3 min) and removal of 80 μl of the supernatant, each aliquot was heated for 3 min to a distinct temperature (37 °C, 40.4 °C, 44 °C, 46.9 °C, 49.8 °C, 52.9 °C, 55.5 °C, 68.6 °C, 62 °C, 66.3 °C) in a PCR machine (Agilent SureCycler 8800), followed by incubation at room temperature (RT) for 3 min. Cells were lysed with 30 μl ice-cold lysis buffer (1.33% NP-40, 2.5 mM MgCl_2_, 1.6× protease inhibitors, 1.6× phosphatase inhibitors, 0.417 U/μl benzonase) on a shaker (500 rpm) at 4 °C for 1 h. Samples were centrifuged at 300*g* for 3 min at 4 °C to remove cell debris, and the supernatant was filtered at 300*g* for 3 min at 4 °C through a 0.45-μm 96-well filter plate (Millipore, MSHVN4550) that was prewetted with plate-wash buffer (0.8% NP-40 in PBS) to remove protein aggregates. Of the flow-through, 25 μl was mixed with 2× sample buffer (180 mM Tris pH 6.8, 4% SDS, 20% glycerol, 0.1 g bromophenol blue) and kept at −20 °C until prepared for MS analysis, while the remainder was used in a bicinchoninic acid assay (Thermo Fisher Scientific), to determine the protein concentration. Samples were diluted to 0.5 μg/μl in 1× sample buffer based on the protein concentrations in the lowest two temperatures (37 °C, 40.4 °C).

### Single-tube TPP with Uniform Progression

Cells were harvested after 1 h of treatment with the compound of interest or DMSO, washed with PBS, and counted. Two aliquots, each containing 10^6^ cells in 100 μl PBS, were distributed over a 96-well PCR plate. After centrifugation (300*g* for 3 min) and removal of 80 μl of the supernatant, one sample was heated to 37 °C for 3 min, followed by incubation at RT for 3 min (“control”), while the other sample was incubated at 37 °C for 5 s, heated to 57 °C at a rate of 0.2 °C/s and then incubated at RT for 3 min (“test”). Cells were transferred to ice and subsequently lysed with 30 μl ice-cold lysis buffer (1.33% NP-40, 2.5 mM MgCl_2_, 1.6× protease inhibitors, 1.6× phosphatase inhibitors, 0.417 U/μl benzonase) and incubated on a shaker (500 rpm) at 4 °C for 1 h. Samples were centrifuged at 300*g* for 3 min at 4°C to remove cell debris, and the supernatant was filtered at 300*g* for 3 min at 4 °C through a 0.45-μm 96-well filter plate (Millipore, MSHVN4550) that was prewetted with plate-wash buffer (0.8% NP-40 in PBS) to remove protein aggregates. Of the flow-through, 25 μl was mixed with 2× sample buffer (180 mM Tris pH 6.8, 4% SDS, 20% glycerol, 0.1 g bromophenol blue) and kept at −20 °C until prepared for MS analysis, while the remainder was used for a bicinchoninic acid assay (Thermo Fisher Scientific) to determine protein concentrations in the control samples. Sample buffer-mixed control samples were diluted to 0.5 μg/μl with a 1:1 ratio of plate wash buffer:2× sample buffer. An equal volume of diluent was added to the test samples.

### MS sample preparation

Proteins were digested as previously described (13). Briefly, for each condition/temperature, 20 μl sample (10 μg protein) was added to 40 μl bead suspension (5% 1:1 Sera-Mag Speed Beads (Thermo Fischer Scientific; 4515-2105-050250 & 6515-2105-050250) prediluted 1:10 in H_2_O, 3.5% formic acid (FA), 70% EtOH) on a 0.45-μm 96-well filter plate (Millipore, MSHVN4550) and incubated on a shaker (500 rpm) for 15 min at RT. Beads were washed four times with 70% ethanol and proteins were digested overnight in 40 μl digest solution (5 mM chloroacetamide, 1.25 mM tris(2-carboxyethyl)phosphine, 200 ng trypsin, and 200 ng LysC in 100 mM Hepes pH 8). After digestion, samples were centrifuged to collect peptides. Peptides were vacuum-dried, reconstituted in 10 μl of water, and labeled for 1 h at RT with either 80 μg of TMT10plex or 50 μg of TMTpro 16plex (Thermo Fisher Scientific) dissolved in 4 μl or 2 μl of acetonitrile (ACN), respectively. An overview of the labels used for each experiment can be found in Table S1). The reaction was quenched with 5 μl of 5% hydroxylamine and samples were combined.

### Sample cleanup, fractionation, and measurement

For TPP, pooled samples were desalted using an OASIS plate (Waters; 186001828BA). After activation with 80% ACN wells were equilibrated with 0.05% FA/water. Samples were loaded and washed twice with 0.05% FA/water. Samples were eluted with 80% ACN and then prepared for high pH fractionation. Offline high-pH reversed-phase fractionation was carried out on an Agilent 1200 Infinity HPLC system, equipped with a Gemini C18 column (3 μm, 110 Å, 100 × 1 mm, Phenomenex) (14). Samples were pooled into 12 fractions. Peptides were separated using an UltiMate 3000 RSLC nano liquid chromatography system (Thermo Fisher Scientific) equipped with a trapping cartridge (Precolumn C18 PepMap 100, 5 μm, 300 μm i.d. × 5 mm, 100 Å) and an analytical column (Acclaim PepMap 100, 75 μm × 50 cm C18, 3 μm, 100 Å). The liquid chromatography system was directly coupled to a Q Exactive Plus mass spectrometer (Thermo Fisher Scientific) using a Nanospray-Flex ion source. Using a 120 min gradient of solvent B (99.9% ACN, 0.1% FA) peptides were eluted and subjected to tandem mass spectrometry (MS/MS). The mass spectrometer was operated in Top10 mode, and dynamic exclusion was applied for 30 s.

For STPP-UP, pooled samples were acidified and desalted using StageTips (15) and eluted with 2× 30 μl of buffer B (80% ACN, 0.01% trifluoroacetic acid). Samples were fractionated using the Pierce High pH Reversed-Phase Peptide Fractionation Kit (Thermo Fisher Scientific) into four fractions and cleaned up using StageTips. Peptides were eluted and applied to reversed-phase chromatography using a nanoLC-Easy1000 coupled online to a Thermo Orbitrap Exploris 480. Using a 120 min gradient of buffer B, peptides were eluted and subjected to MS/MS. The mass spectrometer was operated in Top20 mode and dynamic exclusion was applied for 30 s.

### STPP-UP data analysis

Raw MS files were analyzed using MaxQuant (https://www.maxquant.org/; version 2.1.4.0). Data were searched against either human or mouse UniProt database (downloaded 27-06-2017), with default settings and either TMT10plex or TMTpro 16plex enabled. MaxQuant output files were further processed in R (https://www.r-project.org/; version 4.1.3). Data were filtered for reverse hits, potential contaminants, proteins only identified by site, and protein quantified with less than two unique peptides. Proteins that did not have a signal in all channels were also filtered out. Data were background corrected and normalized by variance stabilizing transformation (*vsn*). Proteins with an adjusted *p*-value <0.05 and a FC >log2(0.5) after differential enrichment analysis using the DEP (16) package (https://bioconductor.org/packages/release/bioc/html/DEP.html; version 1.16.0) were deemed significant. Correction for base differences in protein abundance was done by adding the log2 fold changes between treated and untreated conditions from the control batch to the log2 transformed signal intensities of the control samples from the test batch.

### TPP data analysis

Raw MS files were analyzed using IsobarQuant. Identification of peptide and protein was performed with Mascot against the mouse UniProt database, modified to include known contaminants and the reversed protein sequences. Search parameters: trypsin, missed cleavages 3, peptide tolerance 10 ppm, 0.02 Da for MS/MS tolerance. Fixed modifications were carbamidomethyl on cysteines and TMT10plex on lysine; variable modifications included acetylation on protein N terminus, oxidation of methionine, and TMT10plex on peptide N termini). Output files were loaded into R, merged, filtered for duplicates and proteins with less than two unique peptides and saved in an ExpressionSet R-object. Potential batch effects were removed using *limma* (17), and data were normalized using *vsn* (18). Normalization was done for each temperature independently, to account for the decreasing signal intensity at the higher temperatures. The abundance score of each protein was calculated as the average log2 fold change at the two lowest temperatures (37 °C, 40.4 °C). The stability score of each protein was calculated by subtracting the abundance score from the log2 fold changes of all temperatures and calculating the sum of the resulting values. The significance of abundance and thermal stability scores was assessed using a *limma* analysis, followed by an false discovery rate analysis using the *fdrtool* package.

## Data availability

The MS proteomics datasets have been deposited in the ProteomeXchange Consortium *via* the PRIDE (19) partner repository with the dataset identifier PXD043135 (reviewer login: reviewer_pxd043135@ebi.ac.uk. Password: 8IuPhO2b) and PXD044724 (reviewer login: reviewer_pxd044724@ebi.ac.uk. Password: vMvIAw8g).

## Supporting information

This article contains supporting information (2, 7, 8, 9, 10).

## Conflict of interest

A patent application has been filed by Stichting Oncode Institute for the technology disclosed in this publication. D. W. Z. and M. V. are listed as inventors on the European patent application P134533EP00. The other authors declare that they have no conflicts of interest with the contents of this article.

## Supporting information

Supplemental Table S1

Supplemental Figure S1 *A*, principle behind TPP. Proteins that bind to a compound of interest are more resistant to thermal denaturation. By profiling protein denaturation at different temperatures, we can generate a melting curve that we can compare between two conditions. Shifts in the melting curves and therefore changes in stability are used as a readout for gain or loss of interactions. Figure made with BioRender. *B*, principle behind STPP-UP. Samples are exposed to an incrementally increasing temperature, which will result in an incrementally increasing denaturation rate. Differences in denaturation rate at any temperature in the increment will compound when T_max_ is reached. Figure made with BioRender. STPP-UP, Single-tube TPP with Uniform Progression; TTP, thermal proteome profiling.

Supplemental Figure S2 *A*, time course showing protein denaturation in E14 mESCs at the different temperatures used in the TPP range. Protein abundances were determined by BCA. For each temperature, samples were scaled to their respective untreated (t = 0) samples. Two biological replicates were used per condition. *B*, *boxplots* showing the average Tm-50 in mouse, rat, and human cells. Data were taken from (7, 8, 9, 10). Tm data were filtered for being present in all replicates and between 37 °C and 67 °C. Replicates were averaged. For samples taken from comparative TPP experiments, only the control samples were included. The *boxplots* show the interquartile range (box limits showing the 25th and 75th percentile) and median (*center line*). Whiskers indicate 1.5× the interquartile range. *C*, protein abundances in E14 mESC and LS 174T lysates as determined by BCA after STPP-UP at different T_max_ and ramp rates compared to conventional TPP (37–67 °C). Three biological replicates were used per condition. For STPP-UP, samples were scaled to 37 °C untreated samples. For TPP, samples were normalized to the 37 °C samples. mESC, mouse embryonic stem cell; STPP-UP, Single-tube TPP with Uniform Progression; TTP, thermal proteome profiling.

Supplemental Figure S3 *A*, TPP melting curves for EED in E14 mESCs after 10 μM EED226 or DMSO treatment for 1 h. Data are normalized to the 37 °C sample for each condition. *Shaded regions* show the standard error. Two biological replicates were used per condition. *B*, *scatter plot* showing log2 FC of control and uncorrected test samples from STPP-UP on EED226- and DMSO-treated E14 mESCs. T_max_ was set at 57 °C and ramp rate at 0.2 °C/s. Proteins that are significantly enriched after correction as determined in Figure 2*B* are highlighted by name. Proteins showing mostly enrichment in control samples are highlighted in *blue*, others are highlighted in *red*. mESC, mouse embryonic stem cell; STPP-UP, Single-tube TPP with Uniform Progression; TTP, thermal proteome profiling.

Supplemental Figure S4 *A*, *line plot* showing relative abundance of EED after STPP-UP in R1 mESCs using different T_max_ and ramp rate at 0.2 °C/s. Cells were treated with 10 μM EED226 or DMSO for 1 h (n = 3 biological repicates). For each T_max_, the intensity of the test sample was scaled to the mean intensity of their respective control. *Shaded regions* show the standard error. mESC, mouse embryonic stem cell; STPP-UP, Single-tube TPP with Uniform Progression.

Supplemental Figure S5 *A*, TPP melting curves for DHFR in LS 174T cells after 10 μM C1 or DMSO treatment for 1 h. Data are normalized to the 37 °C sample for each condition. *Shaded regions* show the standard error. Three biological replicates were used per condition. *B*, *scatter plot* showing log2 FC of control and uncorrected test samples from STPP-UP on C1- and DMSO-treated LS 174T cells. T_max_ was set at 57 °C and ramp rate at 0.2 °C/s. Proteins that are significantly enriched after correction as determined in Figure 2*D* are highlighted by name. Proteins showing mostly enrichment in control samples are highlighted in *blue*, others are highlighted in *red*. *C*, *scatter plot* showing log2 FC of control and uncorrected test samples from STPP-UP on C1- and DMSO-treated LS 174T cells. T_max_ was set at 55 °C and ramp rate at 0.1 °C/s. Established C1 targets are highlighted by name. FC, fold change; STPP-UP, Single-tube TPP with Uniform Progression; TTP, thermal proteome profiling.

Supplemental Figure S6 *A* and *B*, *scatter plot* showing log2 fold changes of proteins after treatment of K562 cells with 20 μM staurosporine or DMSO for 1 h and either incremental heating from 37 to 57 °C at a rate of 0.2 °C/s or constant incubation at 57 °C for 3 min (n = 2 biological replicates). Proteins identified as significant (Benjamini–Hochberg corrected *p*-value < 0.05) are highlighted, with (*A*) showing proteins previously identified as (de)stabilizing using TPP (2) and (*B*) showing all human kinases (http://kinhub.org/kinases.html#). Proteins significantly changing using incremental heating are highlighted in *blue*, while proteins significantly changing using constant incubation are highlighted in *red*. Proteins identified as significantly changing in both are highlighted in *dark green*. TTP, thermal proteome profiling.

Supplemental Figure S7 *A*, TPP melting curves for TPM3 in E14 mESCs after 10 μM EED226 or DMSO treatment for 1 h (n = 2 biological replicates). Data are normalized to the 37 °C sample for each condition. *Shaded regions* show the standard error. *B*, *bar plot* for TPM3 showing log2 fold changes for STPP-UP (incremental heating) and single temperature (constant incubation) or stability scores for TPP in E14 mESCs after 10 μM EED226 or DMSO treatment for 1 h. Data on single temperature was taken from the TPP experiment. mESC, mouse embryonic stem cell; STPP-UP, Single-tube TPP with Uniform Progression; TTP, thermal proteome profiling.
