# Supplementary figures and images for: STPP-UP: An alternative method for drug target identification using protein thermal stability
Source: J Biol Chem. 2023 Sep 22;299(11):105279. doi: 10.1016/j.jbc.2023.105279 (PMC10594562; doi:10.1016/j.jbc.2023.105279)

Supplemental figure 1

A

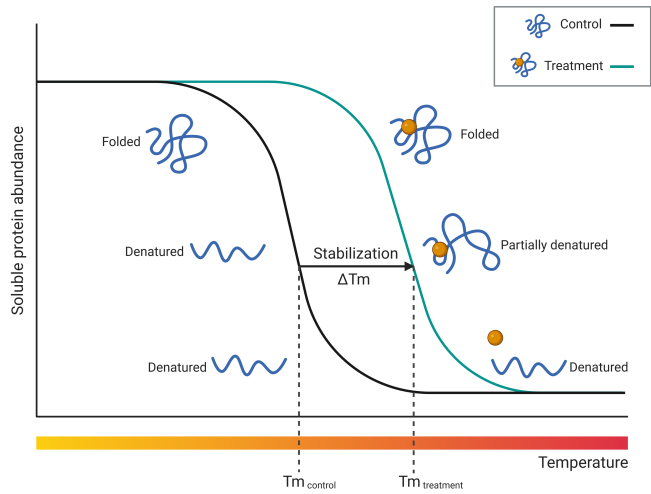

B

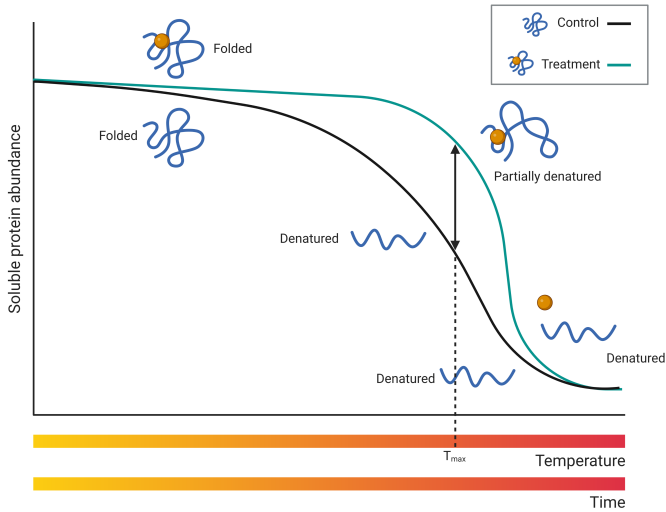

Supplement: Supplemental Figure S1 — A, principle behind TPP. Proteins that bind to a compound of interest are more resistant to thermal denaturation. By profiling protein denaturation at different temperatures, we can generate a melting curve that we can compare between two conditions. Shifts in the melting curves and therefore changes in stability are used as a readout for gain or loss of interactions. Figure made with BioRender. B, principle behind STPP-UP. Samples are exposed to an incrementally increasing temperature, which will result in an incrementally increasing denaturation rate. Differences in denaturation rate at any temperature in the increment will compound when Tmax is reached. Figure made with BioRender. STPP-UP, Single-tube TPP with Uniform Progression; TTP, thermal proteome profiling. [file mmc2.pdf]

Supplemental figure 2

A

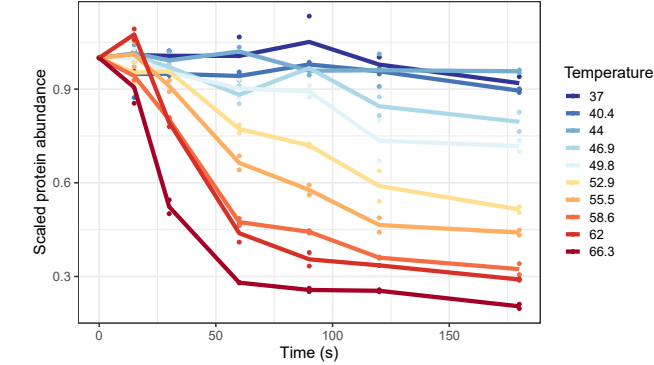

B

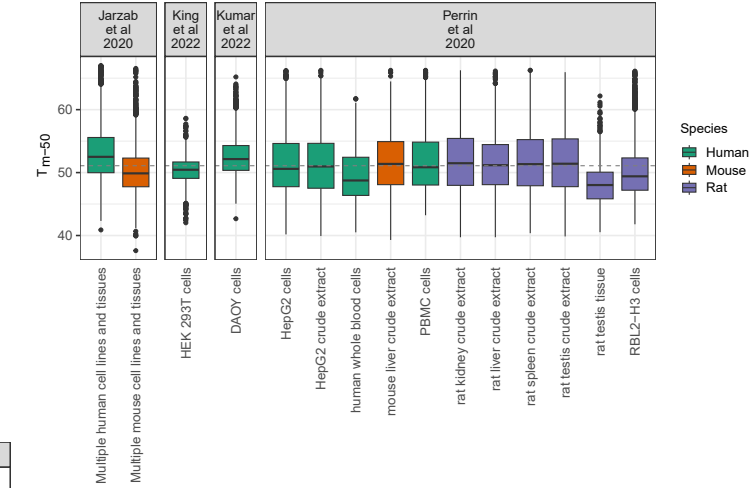

C

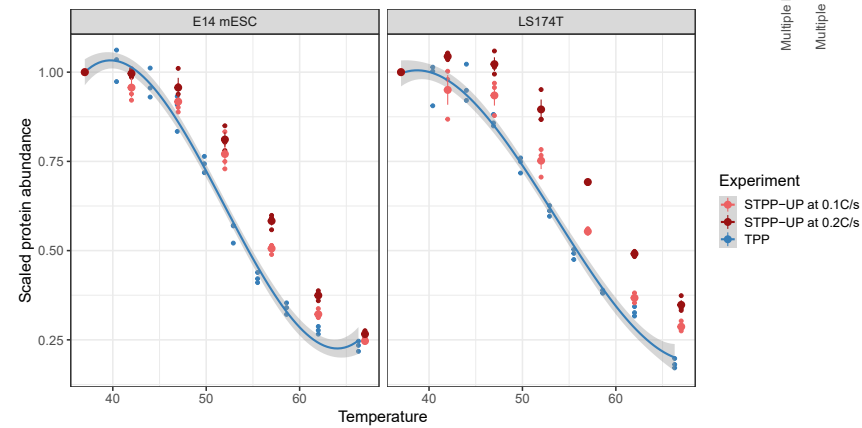

Supplement: Supplemental Figure S2 — A, time course showing protein denaturation in E14 mESCs at the different temperatures used in the TPP range. Protein abundances were determined by BCA. For each temperature, samples were scaled to their respective untreated (t = 0) samples. Two biological replicates were used per condition. B, boxplots showing the average Tm-50 in mouse, rat, and human cells. Data were taken from (7, 8, 9, 10). Tm data were filtered for being present in all replicates and between 37 °C and 67 °C. Replicates were averaged. For samples taken from comparative TPP experiments, only the control samples were included. The boxplots show the interquartile range (box limits showing the 25th and 75th percentile) and median (center line). Whiskers indicate 1.5× the interquartile range. C, protein abundances in E14 mESC and LS 174T lysates as determined by BCA after STPP-UP at different Tmax and ramp rates compared to conventional TPP (37–67 °C). Three biological replicates were used per condition. For STPP-UP, samples were scaled to 37 °C untreated samples. For TPP, samples were normalized to the 37 °C samples. mESC, mouse embryonic stem cell; STPP-UP, Single-tube TPP with Uniform Progression; TTP, thermal proteome profiling. [file mmc3.pdf]

Supplemental figure 3

A

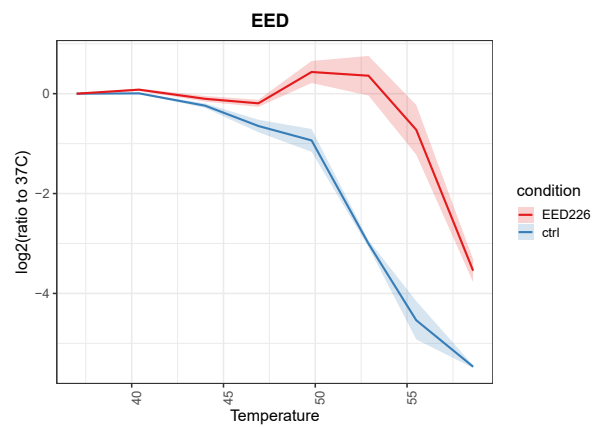

B

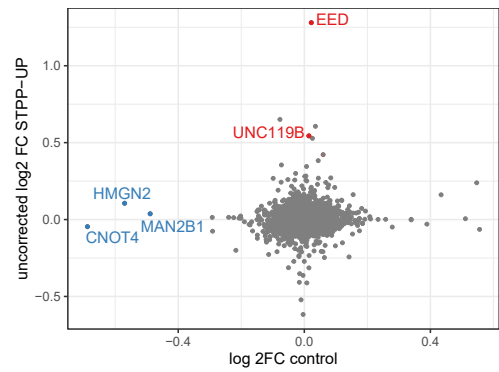

Supplement: Supplemental Figure S3 — A, TPP melting curves for EED in E14 mESCs after 10 μM EED226 or DMSO treatment for 1 h. Data are normalized to the 37 °C sample for each condition. Shaded regions show the standard error. Two biological replicates were used per condition. B, scatter plot showing log2 FC of control and uncorrected test samples from STPP-UP on EED226- and DMSO-treated E14 mESCs. Tmax was set at 57 °C and ramp rate at 0.2 °C/s. Proteins that are significantly enriched after correction as determined in Figure 2B are highlighted by name. Proteins showing mostly enrichment in control samples are highlighted in blue, others are highlighted in red. mESC, mouse embryonic stem cell; STPP-UP, Single-tube TPP with Uniform Progression; TTP, thermal proteome profiling. [file mmc4.pdf]

Supplemental figure 4

A

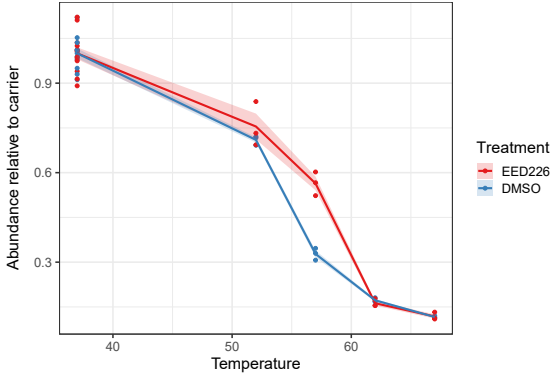

Supplement: Supplemental Figure S4 — A, line plot showing relative abundance of EED after STPP-UP in R1 mESCs using different Tmax and ramp rate at 0.2 °C/s. Cells were treated with 10 μM EED226 or DMSO for 1 h (n = 3 biological repicates). For each Tmax, the intensity of the test sample was scaled to the mean intensity of their respective control. Shaded regions show the standard error. mESC, mouse embryonic stem cell; STPP-UP, Single-tube TPP with Uniform Progression. [file mmc5.pdf]

Supplemental figure 5

A

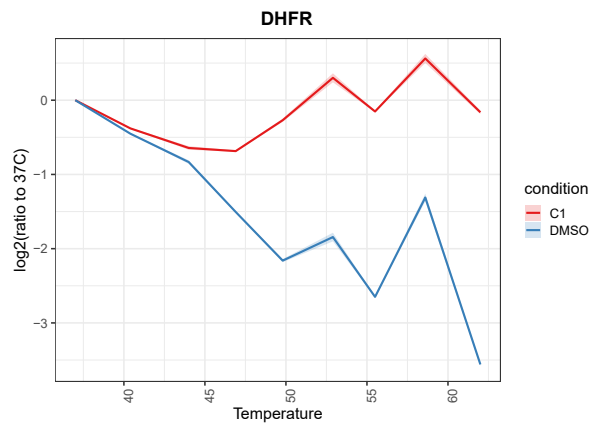

B

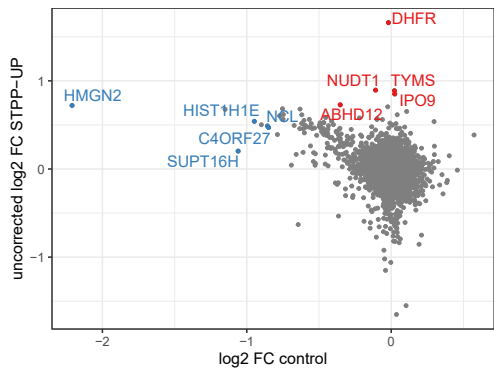

C

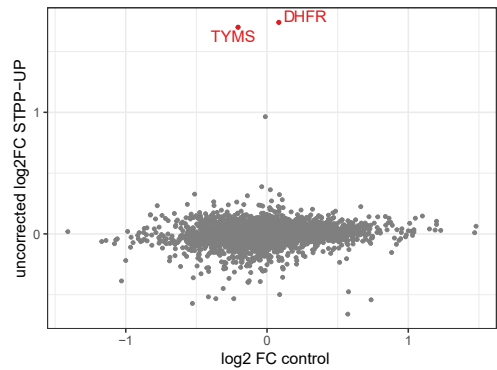

Supplement: Supplemental Figure S5 — A, TPP melting curves for DHFR in LS 174T cells after 10 μM C1 or DMSO treatment for 1 h. Data are normalized to the 37 °C sample for each condition. Shaded regions show the standard error. Three biological replicates were used per condition. B, scatter plot showing log2 FC of control and uncorrected test samples from STPP-UP on C1- and DMSO-treated LS 174T cells. Tmax was set at 57 °C and ramp rate at 0.2 °C/s. Proteins that are significantly enriched after correction as determined in Figure 2D are highlighted by name. Proteins showing mostly enrichment in control samples are highlighted in blue, others are highlighted in red. C, scatter plot showing log2 FC of control and uncorrected test samples from STPP-UP on C1- and DMSO-treated LS 174T cells. Tmax was set at 55 °C and ramp rate at 0.1 °C/s. Established C1 targets are highlighted by name. FC, fold change; STPP-UP, Single-tube TPP with Uniform Progression; TTP, thermal proteome profiling. [file mmc6.pdf]

Supplemental figure 6

A

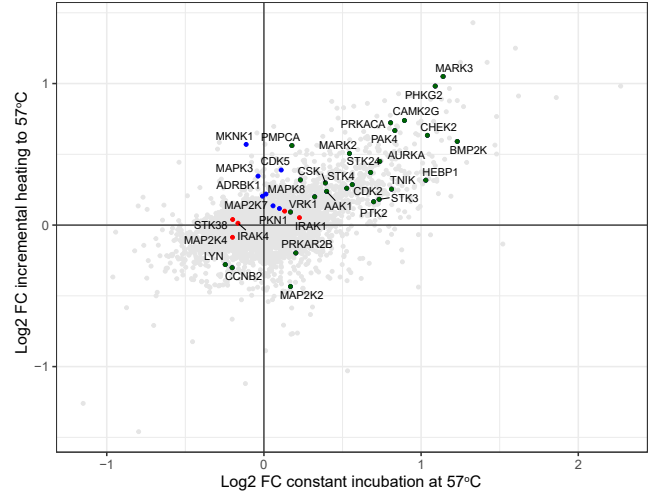

B

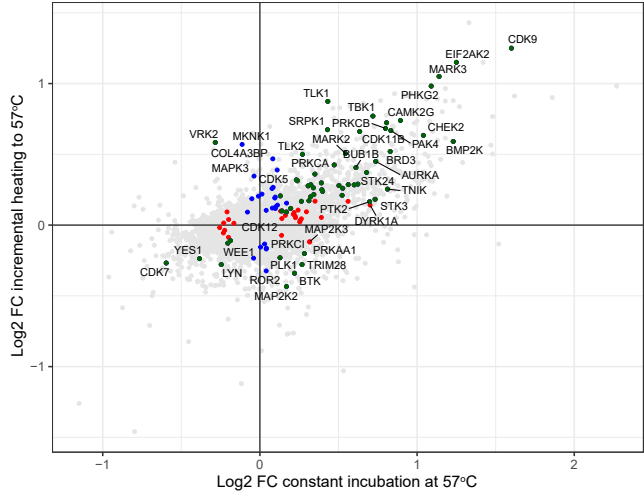

Supplement: Supplemental Figure S6 — A and B, scatter plot showing log2 fold changes of proteins after treatment of K562 cells with 20 μM staurosporine or DMSO for 1 h and either incremental heating from 37 to 57 °C at a rate of 0.2 °C/s or constant incubation at 57 °C for 3 min (n = 2 biological replicates). Proteins identified as significant (Benjamini–Hochberg corrected p-value < 0.05) are highlighted, with (A) showing proteins previously identified as (de)stabilizing using TPP (2) and (B) showing all human kinases (http://kinhub.org/kinases.html#). Proteins significantly changing using incremental heating are highlighted in blue, while proteins significantly changing using constant incubation are highlighted in red. Proteins identified as significantly changing in both are highlighted in dark green. TTP, thermal proteome profiling. [file mmc7.pdf]

Supplemental figure 7

A

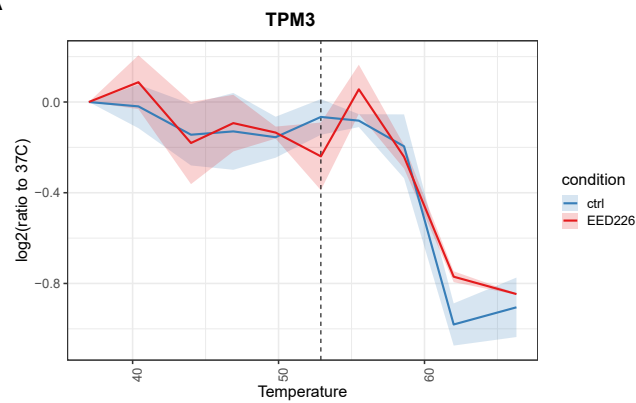

B

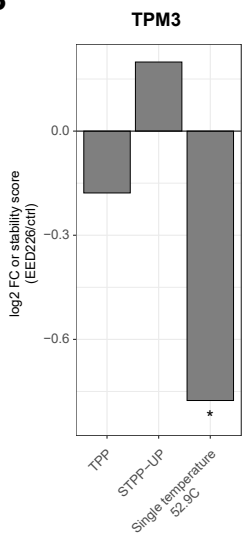

Supplement: Supplemental Figure S7 — A, TPP melting curves for TPM3 in E14 mESCs after 10 μM EED226 or DMSO treatment for 1 h (n = 2 biological replicates). Data are normalized to the 37 °C sample for each condition. Shaded regions show the standard error. B, bar plot for TPM3 showing log2 fold changes for STPP-UP (incremental heating) and single temperature (constant incubation) or stability scores for TPP in E14 mESCs after 10 μM EED226 or DMSO treatment for 1 h. Data on single temperature was taken from the TPP experiment. mESC, mouse embryonic stem cell; STPP-UP, Single-tube TPP with Uniform Progression; TTP, thermal proteome profiling. [file mmc8.pdf]
